# Draft Genome Sequence of *Methylophaga lonarensis* MPL^T^, a Haloalkaliphilic (Non-Methane-Utilizing) Methylotroph

**DOI:** 10.1128/genomeA.00202-13

**Published:** 2013-05-09

**Authors:** Sudarshan A. Shetty, Nachiket P. Marathe, Hitendra Munot, Chakkiath Paul Antony, Dhiraj P. Dhotre, J. Colin Murrell, Yogesh S. Shouche

**Affiliations:** Microbial Culture Collection, National Centre for Cell Science, Ganeshkhind, Pune, Maharashtra, Indiaa;; School of Environmental Sciences, University of East Anglia, Norwich, United Kingdomb

## Abstract

*Methylophaga lonarensis* strain MPL^T^ is a haloalkaliphilic methylotroph isolated from Lonar Lake, a saline and alkaline lake in Maharashtra, India. Strain MPL^T^ utilizes methanol as its sole carbon and energy source. Here, we present the draft genome sequence of *M. lonarensis* MPL^T^ (VKM B-2684^T^ = MCC 1002^T^).

## GENOME ANNOUNCEMENT

Members of the genus *Methylophaga* are known for their ability to utilize one-carbon compounds (C_1_) and have been isolated mostly from habitats of low water activity (1). Four previously described genome sequences of *Methylophaga* spp. have provided insights into the metabolic potential of marine *Methylophaga* strains (2–4).

*Methylophaga lonarensis* strain MPL^T^ was isolated from Lonar Lake, a saline and alkaline ecosystem that is located in Buldhana district, Maharashtra, India (5). Strain MPL^T^ is a moderately haloalkaliphilic methylotroph that assimilates carbon via the ribulose monophosphate (RuMP) pathway. *Methylophaga* spp. were previously shown to actively assimilate methanol in the Lonar Lake sediments (6). The genome of *M. lonarensis* was sequenced in order to gain a better understanding of the genetic potential of haloalkaliphilic methylotrophs.

The draft genome of *M. lonarensis* MPL^T^, determined by a whole-genome shotgun approach using Ion torrent PGM (200-bp single-end shotgun sequencing, 1,572,266 assembled reads), is 2,637,625 bp with coverage of 80.7×. Assembly of sequencing reads was done using MIRA assembler v3.4.0 with the default parameters (7). After assembly, a total of 116 contigs were obtained with a mean G+C content of 49.7%, which is in agreement with experimental data (G+C content of 50.0% [5]). Annotation was done using RAST server, an automated annotation system (8).

The chromosome of *M. lonarensis* MPL^T^ harbors 2,614 protein-coding genes. Fifty-five RNA genes, with six rRNA operons (four 5S rRNA and one each for 23S and 16S rRNA), were identified using RNAmmer v1.2 (9). Twenty-five genes encoding putative cytochrome *bc*_1_ complex and three genes for terminal oxidase cbb3 were found. The gene clusters responsible for methanol oxidation (*mxaFJDIACKL*) were identified. Genetic potential for osmoregulation was confirmed by the presence of genes encoding enzymes associated with ectoine biosynthesis and sucrose accumulation. Genes involved in pyrroloquinoline quinone biosynthesis (*pqqBCDE*) and genes encoding methylamine dehydrogenase (*mauABDEF*) were identified.

Genes encoding enzymes of the Entner-Doudoroff variant of the RuMP pathway, the tricarboxylic acid (TCA) cycle, the pentose phosphate pathway, sulfur metabolism, and nitrogen metabolism were detected. Key genes associated with the serine- or the ribulose-bisphosphate-pathway of C_1_ assimilation were absent, in agreement with enzyme assay data (5). Extrachromosomal elements, such as phages, prophages, and plasmids, were not detected in the draft genome. Future in-depth analysis of the *M. lonarensis* MPL^T^ genome along with other *Methylophaga* genomes will shed light on the biotechnological potential of these methylotrophs and also help to understand how they adapt to environments of low water activity.

### Nucleotide sequence accession numbers.

This whole-genome shotgun project has been deposited at GenBank under the accession no. APHR00000000. The version described in this paper is the first version, accession no. APHR01000000.
